# The influence of proactive personality on students’ knowledge sharing: the chain mediating effect of class climate and learning engagement

**DOI:** 10.3389/fpsyg.2024.1487345

**Published:** 2024-12-24

**Authors:** Ye Lin, Yongchao Jin, Rui Yang

**Affiliations:** ^1^School of Psychology and Mental Health, North China University of Science and Technology, Tangshan, China; ^2^School of Philosophy and Sociology, Jilin University, Changchun, China

**Keywords:** proactive personality, knowledge sharing, class climate, learning engagement, chain mediating effect, student

## Abstract

**Introduction:**

Knowledge sharing is an effective means of knowledge management in colleges and universities, which is of great significance for improving the quality and efficiency of universities and enhancing the balanced development of educational resources. The present study investigated the influence students’ proactive personalities drive knowledge-sharing activities, and examined the significance of class climate and learning engagement as mediating factors, utilizing the perspectives of social exchange theory (SET) and the job demands and resources model (JD-R) .

**Methods:**

A convenience sampling method was employed to survey 1,053 Chinese college students, and evaluated them using the Proactive Personality Scale (PPS), Learning Engagement Scale (LES), Class Climate Scale (CCS), and Knowledge Sharing Behavior Scale (KSBS). The structural equation model and bootstrap method to explore the significance of the mediating effect of class climate and learning engagement between proactive personality and knowledge sharing of college students.

**Results:**

(1) Proactive personality was positively associated with students’ knowledge sharing behavior; (2) Class climate had a significant mediating effect between proactive personality and knowledge sharing behavior of college students, and the mediating effect of class climate is 21.2%; (3) Learning engagement mediated effect between proactive personality and knowledge sharing behavior, and the mediating effect ratio of learning engagement is 8.4%; (4) Class climate and learning engagement play a chain mediating role in the relationship between proactive personality and knowledge sharing behavior of students, and the chain mediating role accounted for 2.1% of the total effect.

**Conclusion:**

Proactive personality exerts a significant impact on students’ knowledge-sharing behaviors. However, this effect is not direct; it is mediated by both class climate and learning engagement. Specifically, students exhibiting higher levels of proactivity are more likely to actively engage in the learning process and foster a positive class climate, which, in turn, enhances their propensity to share knowledge with peers.

## 1 Introduction

In the current era of knowledge economy, knowledge (intellectual capital) is gradually regarded as the most important resource of social and economic activities (Alavi and Leidner, 2001; Cabrera et al., 2006; Fan and Beh, 2024). The augmentation of knowledge’s potency becomes evident through its active dissemination and sharing among individuals and entities (Nonaka and Takeuchi, 1995). Knowledge sharing (KS) has been widely acknowledged as a fundamental pillar underpinning numerous knowledge management programs (Hislop, 2013), representing an essential cornerstone in facilitating organizational learning and innovation. In a workplace setting, effective knowledge sharing (KS) has been observed to yield benefits such as enhanced learning, troubleshooting, creativity, decision-making, ingenuity, and individual and institutional performance (Hussain et al., 2017). However, within the context of higher education institutions (HEIs), KS plays an even more vital role, surpassing its importance in other types of organizations (Al-Kurdi et al., 2020). This is primarily attributed to the fundamental mission of HEIs, which involves the creation and dissemination of knowledge through research, teaching, and learning endeavors (Al-Kurdi et al., 2018; Iqbal, 2021). Numerous studies have been conducted to explore the factors that impact knowledge sharing (KS) in diverse organizational contexts (Bock et al., 2005; Wei et al., 2009). Few studies have specifically investigated knowledge sharing (KS) in universities, with a particular on the KS among students. However, knowledge sharing can be intricate due to the potential conflicts it may pose with personal interests (Matzler et al., 2008). Consequently, it becomes imperative to delve deeper into comprehending the underlying elements that can influence students’ to engage in KS is crucial.

Although there has been a plethora of studies concerning knowledge sharing, there are still several sizable research gaps. Of the various factors that impact knowledge sharing, personality has received considerable attention (e.g., Yin et al., 2023; Obrenovic et al., 2022). A plethora of research has demonstrated that proactive personality may serve as a unique and incremental predictor of job performance beyond Big Five personality traits (e.g., Thomas et al., 2010). As defined by Bateman and Crant (1993), proactive personality refer to “an individual’s propensity to initiate environmental changes”. As a stable and cross-situation personality type, proactive personality makes individuals show obvious proactive behavior. Individuals with this personality trait are less restricted by situational pressure and can make changes to the environment by exerting their own initiative. Furthermore, individuals with high levels of proactive personality also tend to have elevated levels of role breadth self-efficacy, leading them to engage in more positive, interpersonal, and comprehensive beneficial organizational behaviors such as knowledge sharing (Jiang et al., 2021). Despite the importance of proactive personality in relation to knowledge sharing behavior, research on the topic remains limited, and the mechanisms and conditions underlying this relationship have yet to be systematically verified. Some studies suggest that the influence of proactive personality on knowledge sharing is not context-dependent, as individuals with this personality type possess core self-evaluations that enable them to maintain high-quality interactions with superiors and colleagues in any given context, thus contributing to extra-role behaviors such as knowledge sharing (Abbas et al., 2018). However, other research suggests that the role of proactive personality varies across different situations. In situations with high autonomy and low organizational behavior requirements, individuals with high proactive personality are more inclined to knowledge sharing (Lv et al., 2018). As for the group of college students, they may suffer from the loss of scholarships, honorary titles and postgraduate admission places due to the one-way output of knowledge, or they may get negative feedback and affect their self-image because of the uneven quality of shared knowledge. As a result, in practice, students may choose to wait and see or remain silent rather than take the initiative to share knowledge. Therefore, it is essential to further investigate the influencing mechanisms and conditions associated with proactive personality and knowledge sharing among students.

The Job Demands-Resources (JD-R) model, initially proposed by Demerouti et al. (2001), is an eminent theoretical framework that assesses the impact of job demands and resources on employee well-being and workplace outcomes (Li et al., 2023). According to job demands and resources (JD-R) model, the work context encompasses two crucial categories of resources that facilitate meeting work-related requirements: individual resources (positive attitude, proactive personality, self-efficacy, etc.) and work-related resources (social support, growth opportunities, organizational climate, etc.) (Bajaba et al., 2021; Lucal et al., 2020). As a key individual resource, proactive personality can promote and guide individual change and self-motivation, thereby promoting the willingness to actively acquire work-related resources through increased work engagement (Sun and Yoon, 2022). As a positive personality trait, proactive personality is the driving factor of individual initiative (Campbell, 2000). For instance, individuals exhibiting high proactive personality levels proactively aim to influence their environment and actively surmount environmental barriers to acquire additional resources that can be leveraged in problem-solving (Bateman and Crant, 1993). Moreover, research conducted in the realm of learning suggests that individuals possessing high initiative exhibit more proactive learning behaviors and enhanced engagement levels throughout the learning process (Bao et al., 2022). Simultaneously, the class learning climate, functioning as a work resource environment, embodies students’ collective perception of the available learning resources within educational settings, manifesting through the sense of belonging, learning atmosphere, and equitable ambiance. The higher the evaluation of the class climate, the more supportive resources individuals perceive in the environment for their learning needs, and the more positive their relationship with the group becomes. According to the social exchange theory (Emerson, 1976), individuals tend to engage in cooperative behaviors to enhance overall class learning and facilitate knowledge sharing when establishing a psychological contract with their classmates based on social-emotional exchanges. In light of these considerations, the present study aims to explore how students’ proactive personalities drive knowledge-sharing activities, and how the social and emotional aspects of the classroom environment (class climate) and students’ engagement with the learning process (learning engagement) mediate this effect, utilizing the perspectives of social exchange theory (SET) and the job demands and resources model (JD-R).

## 2 Literature review

### 2.1 Proactive personality and knowledge sharing

Knowledge sharing refers to the dissemination of task-related knowledge, experiential insights, skill sets, pedagogical approaches, innovative ideation, or implementing procedures to help others and to collaborate to solve problems (Yao et al., 2024). Knowledge sharing can take place through multiple modalities, encompassing the exchange of written information, engaging in face-to-face conversations, establishing networks with proficient experts, as well as the documentation, systematization, and preservation of knowledge to facilitate accessibility for others (Cummings, 2004; Im et al., 2023). It is observed that individuals who display a propensity toward knowledge sharing demonstrate a willingness to disseminate their expertise and insights among their colleagues. In practice, however, an issue that frequently arises is the reticence of numerous organizational members to impart their knowledge to their peers (Shi et al., 2023), and in some instances, even deliberately conceal or withhold valuable knowledge (Connelly et al., 2012). The complexity of knowledge sharing is amplified when it is linked with individual gains, particularly for limited resources such as students’ scholarships, honorary titles, and recommended postgraduate degree, leading to a substantial decline in the willingness of individuals to share (Davenport and Prusak, 1998). Therefore, it is imperative that research on knowledge sharing shifts its focus to the “human” element as the active participant in knowledge sharing initiatives. Previous research has revealed that individuals who possess proactive personality traits exhibit enhanced flexibility and are less constrained by environmental factors, which allows them to adapt quickly and influence environmental changes. Such individuals also excel in identifying opportunities, taking decisive action, and persisting until successful outcomes are achieved. Proactive personality traits have been shown to have a positive impact on individual career success, job satisfaction, organizational performance, and innovation behavior within organizations (Haynie et al., 2020). Individuals characterized by high levels of proactive personality also display a heightened degree of socialization, actively seek constructive feedback, engage in creative activities, and undertake more out-of-role behaviors by redefining work roles to enhance the value of their work (Crant, 2000). Based on the job demand-resource theory, individuals with a proactive personality exhibit a greater propensity for engaging in knowledge sharing during various learning activities, such as formulating learning plans, adjusting learning strategies, and accessing support resources. Students achieve the conversion of individual knowledge into organizational knowledge through various mechanisms including individual-level demonstration, group-level dialogue and communication, and overall level integration. Consequently, students’ knowledge sharing can be seen as an active process of resource exchange, encompassing both knowledge contribution and acquisition. Notably, students characterized by proactive personality traits demonstrate proficiency in both contributing their own knowledge and acquiring additional knowledge resources throughout this process. Accordingly, the following hypothesis was formulated:

Hypothesis 1. (H1): Proactive personality has significant positive influence on students’ knowledge sharing.

### 2.2 The mediating role of class climate

The concept of “class climate” refers to the collective perceptions and experiences of students within a classroom setting regarding various aspects, including the fairness of grading practices, the level of support received from teachers and peers, the perceived cohesion among classmates, and the presence of competitive elements (Wang and Jiang, 2023; Koth et al., 2008; Thapa et al., 2013; Zullig et al., 2010). In the field of organizational psychology, class climate is considered to be influenced by organizational climate, which refers to how members of an organization subjectively perceive their organizational environment. An organizational environment that is characterized by innovation, fairness, and congeniality engender cultivate an climate that is open, tolerant, novel, and respectful, thereby facilitating active knowledge and skill exchange among its constituents. Individuals have varying inclinations to exert influence on their environment. The proactive personality, characterized by traits such as self-disclosure, heightened positive emotional states, adaptability in interpersonal interactions, and active communication and collaboration with others, has a beneficial impact on fostering a conducive class climate. Individuals with a proactive personality demonstrate greater initiative, adaptability to their surroundings, and exert a positive influence on their environment. Moreover, a number of empirical studies have revealed a strong relationship between organizational climate and the willingness to share knowledge (Chennamaneni et al., 2012). Jones (2022) delved into the intricate interplay between the working environment, organizational democracy, and the willingness to share knowledge. The research findings demonstrate that individuals within an organization exhibit the highest inclination for knowledge sharing when they are situated in a favorable working environment and are actively involved in organizational management (Jones, 2022). Similarly, the study of Hinds and Pfefer shows that when the organization is in an climate of high trust for individuals and organizations, the organization’s willingness to share knowledge is stronger (Hinds and Pfeffer, 2003). Accordingly, the following hypothesis was formulated:

Hypothesis 2. (H2): Class Climate plays a mediating role in the influence of proactive personality on students’ knowledge sharing.

### 2.3 The mediating role of learning engagement

The concept of learning engagement is transformed through work engagement, Schaufeli et al. (2002) extended the study of work engagement to students and expounded the concept of learning engagement. Schaufeli and Bakker (2004) describes learning engagement as a positive, fulfilling state of mind associated with learning, characterized by energy, dedication, and focus. To evaluate learning engagement within the context of higher education, Schaufeli et al. (2002) have developed a comprehensive scale that assesses three distinct dimensions: vigor, dedication, and absorption. Vigor is a construct that pertains to an individual’s ability to sustain high levels of energy and mental resilience while performing tasks, driven by a proactive disposition toward investing effort and demonstrating persistence in the face of adversities. Dedication can be defined as an intense involvement in one’s work, accompanied by a profound sense of significance, enthusiasm, inspiration, pride, and the desire to tackle challenges head-on. Finally, absorption is characterized by a state of complete concentration and joyful immersion in work tasks, wherein time seems to elapse rapidly, and one has difficulties detaching oneself from work (Schaufeli et al., 2006). These three dimensions comprehensively encompass all facets of learning engagement, including behavioral manifestations, emotional and affective aspects, as well as cognitive involvement, providing a holistic framework for assessing the learning engagement (Fredricks, 2016).

In general, individuals exhibiting high levels of learning engagement demonstrate greater willingness and positive behavior toward knowledge sharing. Specifically, one is that individuals will experience more positive emotions in learning engagement, which are considered an important driver of altruistic behavior. Second, individuals who display elevated levels of learning engagement are more likely to garner increased trust and support from teachers and classmates. This trust, in turn, facilitates opportunities for students to showcase their abilities, thereby driving participation in scientific research, learning cooperation, and innovative scientific pursuits (Zhang et al., 2023). Additionally, this group recognition also cultivates a sense of responsibility and mission among individuals, motivating them to contribute to the class and promote knowledge sharing. Third, when individuals exhibit highly engaged learning behavior, characterized by prolonged focus on the learning task and increased investment of resources, a notable enhancement in their learning role identity is observed. Based on role identity theory, individuals actively align their behavior with the requirements and expectations of their assigned roles, thereby demonstrating proactive learning behaviors that are consistent with their role cognition. Consequently, there is an increasing emphasis on collaborative learning strategies that promote innovative approaches to learning, particularly knowledge sharing, rather than the tendency to withhold or conceal knowledge. Furthermore, Rizzotto highlighted the sensitivity of students to the school climate, which not only influences their behavior and adaptation but also significantly impacts their learning outcomes (Rizzotto, 2022). Supporting this notion, Reyes et al. (2012) suggest that a high-quality classroom climate has a positive impact on students’ level of engagement in the learning process. Accordingly, the following hypothesis was formulated:

Hypothesis 3. (H3): Learning engagement plays a significant mediating role in the relationship between proactive personality and knowledge sharing of college students.

Hypothesis 4. (H4): Class climate and Learning engagement play a chain mediating role between the relationship of proactive personality and students’ knowledge sharing.

**FIGURE 1:**
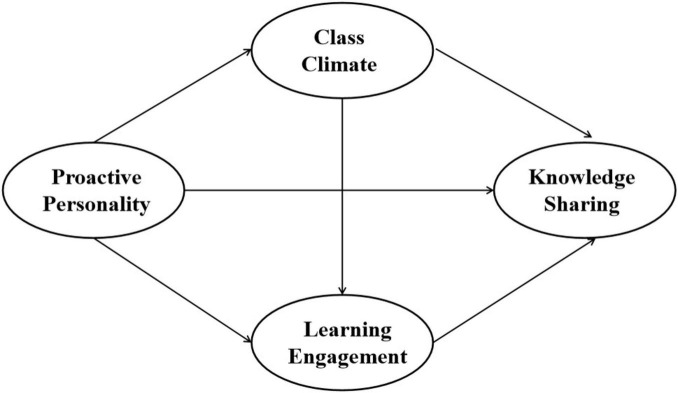
Theoretical research framework.

In summary, this study derives a theoretical framework for the impact of proactive personality on students’ knowledge sharing, with class climate and learning engagement as mediating variables (Figure 1).

## 3 Materials and methods

### 3.1 Data collection and sampling

In this study, a convenience sampling method was employed to recruit participants from two universities, North China University of Science and Technology and Tangshan College, both of which are located in Hebei province in northern China. The instructions and questionnaires were disseminated via an online platform known as “wenjuanxing”, which is a popular, powerful, and personalized questionnaire design system. Data collection spanned from December 27, 2023 to January 27, 2024. Prior to participation, all participants were informed of the aims, and measures were taken to ensure authenticity and anonymity. Hence, the information that could identify individual participants can’t be accessed. Upon agreement, participants gained access to the questionnaire filling interface by scanning a designated QR code and enabling them to indicate their responses by selecting their choices. Finally, 1,067 questionnaires were received, out of which 1,053 were deemed eligible based on inclusion criteria, resulting in an effective response rate of 98.7%. The data of this study are not openly available but can be made available by the corresponding author upon reasonable request.

The demographic profile of the participants revealed that 540 male respondents (51.3%) and 513 female respondents (48.7%) took part in the study. The distribution of academic year consisted of 596 freshmen (56.6%), 276 sophomores (26.2%), 129 juniors (12.3%), and 52 seniors (4.9%). With regards to their post-graduation plans, 658 students (62.5%) expressed their intent to pursue further studies, while 148 students (14.1%) planned to enter the workforce. Additionally, 25 students (2.4%) had alternative plans, and 222 students (21.1%) remained undecided about their future after graduation. Regarding academic performance, 51 participants (4.8%) demonstrated poor performance, 120 participants (11.4%) exhibited lower than average performance, 510 participants (48.4%) maintained an average level of performance, 276 participants (26.2%) showcased above-average performance, and 96 participants (9.1%) achieved good academic standing. Furthermore, 496 students (47.1%) had previous experience serving as class leaders or community leaders, while 557 students (52.9%) had not assumed such leadership roles.

### 3.2 Measurement

The measurement system of this paper includes four scales: proactive personality scale, knowledge sharing scale, learning engagement scale and class climate scale. In order to ensure the reliability and validity of the scale, all measurement scales of the present study were adopted from previous studies.

#### 3.2.1 Proactive personality scale (PPS)

The proactive personality scale compiled by Bateman and Crant (1993) and revised by Shang and Gan (2009) was used to assess proactive personality characteristics in this study. The scale consists of 11 items with 7-point Likert-type responses, ranging from 1 (completely disagree) to 7 (completely agree), such as “If I see someone in trouble, I help out in any way I can.” Elevated scores on this scale are indicative of heightened levels of proactive personality traits. The internal consistency of the proactive personality scale was assessed using Cronbach’s alpha coefficient, yielding a value of 0.93 in this study, signifying a high level of internal reliability.

#### 3.2.2 Learning engagement scale (LES)

The learning engagement scale compiled by Schaufeli and Bakker (2004) contains three dimensions of motivation, energy and concentration, with a total of 17 questions ranging from “never” to “Always” is rated from 1 to 7. An elevated score corresponds to a heightened level of learning engagement. The original scale exhibits superior structural validity, and the internal consistency reliability of both the overall scale and its subscales surpasses that of the original scale’s value of 0.75. In the present study, the scale had a good consistency, as indicated by a Cronbach’s coefficient of 0.94.

#### 3.2.3 Class climate scale (CCS)

The class climate scale is adapted from the «College Students’Class Psychological Atmosphere Evaluating Scale»compiled by Li and Chen (2014), with 19 items in total. One sample item is “Our class is very united.” Participants respond to the items on a 7-point Likert-type scale ranging from 1 (completely disagree) to 7 (completely agree), with higher scores indicating elevated levels of class climate. The class climate scale exhibited a high degree of internal consistency in this study, as indicated by a Cronbach’s alpha coefficient of 0.97.

#### 3.2.4 Knowledge sharing behavior scale (KSBS)

Students’ knowledge sharing behavior was evaluated using a 10-item scale developed by Zhang et al. (2008). The scale comprises 10 items with 7-point Likert-type responses, ranging from 1 (completely disagree) to 7 (completely agree). A representative item was, “When other students have problems in their study, i will give explanations or answers.” Participants provided responses based on their personal experiences. Higher scores on the scale indicate a greater level of knowledge sharing. The internal consistency reliability of the knowledge sharing behavior scale was high in this study, with a Cronbach’s alpha coefficient of 0.94.

#### 3.2.5 Common method bias

Using the Harman one-way method, a common method bias test was conducted on a set of 62 questions with four variables. The exploratory factor analysis showed that the first factor accounted for 21.75% of the variance, which is less than half of the critical criterion of 69.88%. The findings indicate the absence of significant common method variation in the data, thereby establishing its suitability for further examination and evaluation.

### 3.3 Data analysis

The statistical analysis in this study was conducted using SPSS 22.0 and Mplus 8.3 software. First, descriptive statistics were carried out using SPSS to obtain the means and standard deviations for each dimension. Pearson correlation tests were subsequently employed to examine the interrelationships among the variables. The study then utilized structural equation modeling to explore the associations between the four variables of proactive personality, knowledge sharing behavior, class climate, and learning engagement. Finally, the bias-corrected non-parametric percentile Bootstrap method with 5000 random replicates was employed to determine the 95% confidence intervals. This approach facilitated an investigation of the chain intermediary role of class climate and learning engagement in the relationship between proactive personality and students’ knowledge sharing behavior.

## 4 Results

### 4.1 Correlations and descriptive statistics

Table 1 presents the results of descriptive statistics and correlation tests for the variables under investigation. Notably, proactive personality exhibited a significant and positive correlation with students’ Knowledge Sharing Behavior (*r* = 0.73, *p* < 0.01). Furthermore, students’ Knowledge Sharing Behavior demonstrated significant and positive correlations with both learning engagement (*r* = 0.59, *p* < 0.01) and class climate (*r* = 0.64, *p* < 0.01). These findings from the correlation tests provide support for the subsequent analysis of the structural equation model.

**TABLE 1:** Means, standard deviations and correlations among variables in the research.

Variable	1	2	3	4
Proactive personality	–			
Knowledge Sharing	0.73**	–		
Learning engagement	0.59**	0.59**	–	
Class climate	0.50**	0.64**	0.50**	–
M	5.2	5.33	4.86	5.2
SD	1.15	1.16	1.1	1.26

**p < 0.01.

### 4.2 Structural equation model path verification

**FIGURE 2:**
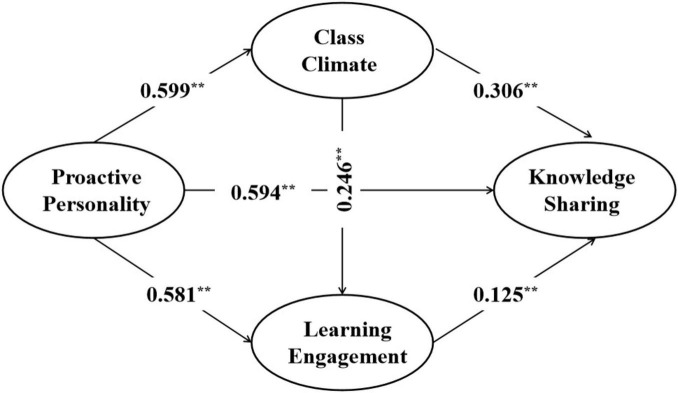
Structural equation model path result. **p < 0.01.

To test the impact of sustainable teaching innovation on graduate students’ creative thinking, a structural equation model was developed with good model fit (X2/df = 4.52, CFI = 0.907, TLI = 0.902, RMSEA = 0.058). The results of the structural equation model path analysis are presented in Figure 2. The findings demonstrate that proactive personality exhibited a significant and positive impact on class climate (β = 0.599, *p* < 0.05), learning engagement (β = 0.581, *p* < 0.05), and knowledge sharing (β = 0.594, *p* < 0.05). Furthermore, class climate (β = 0.306, *p* < 0.05) and learning engagement (β = 0.125, *p* < 0.05) were found to significantly and positively influence students’ knowledge sharing. Notably, class climate emerged as a significant and positive mediating variable for learning engagement (β = 0.246, *p* < 0.05), thus providing support for hypothesis H1. However, whether and to what extent the independent and chain mediating effects of class climate and learning engagement are true remains to be further explored.

### 4.3 Mediation effect test

To assess the potential mediating role of class climate and learning engagement, a bias-corrected non-parametric percentile Bootstrap method was employed (Wen et al., 2010). As illustrated in Table 2, the analysis revealed a total effect of 0.869 (*Z* > 1.96, 95% CI = [0.770, 0.973]) for Proactive personality on students’ knowledge sharing. Additionally, the total indirect effect was estimated to be 0.275 (*Z* > 1.96, 95% CI = [0.210, 0.346]). Consequently, the percentage of the overall indirect effect accounted for 31.6% of the total effect.

**TABLE 2:** Mediating results in the SEM.

Mediation effect test	Estimation	BootStrap SE	*Z*	Bootstrapping 95% CI		*p*	Mediation effect proportion
				**Lower**	**Upper**		
Total effect	0.867	0.048	18.135	0.775	0.963	–	–
Direct effect	0.601	0.053	11.360	0.499	0.705	–	–
Indirect effect	0.265	0.032	8.272	0.205	0.331	0.000	30.6%
Indirect effect(PP → CC → KS)	0.188	0.024	7.993	0.145	0.238	0.000	21.7%
Indirect effect(PP → LE → KS)	0.061	0.020	3.034	0.024	0.103	0.002	7.0%
Indirect effect(PP → CC → LE → KS)	0.017	0.006	2.565	0.006	0.031	0.010	2.0%

With respect to the influence of each mediating variable, our analysis revealed an indirect effect of 0.184 (*p* < 0.05, 95% CI = [0.141, 0.236]) for class climate and an indirect effect of 0.073 (*p* < 0.01, 95% CI = [0.034, 0.118]) for learning engagement. The chain mediating effect through class climate to learning engagement was estimated at 0.018 (*p* < 0.01, 95% CI = [0.008, 0.035]). Notably, the proportion of the total indirect effect attributed to class climate accounted for 21.2%, while the proportion for learning engagement stood at 8.4%. Additionally, the proportion of the indirect effect attributed to chain mediation was 2.1%. Overall, these findings provide support for hypotheses H2, H3, and H4, which propose the existence of mediating and chain mediating effects for class climate and learning engagement.

## 5 Discussion

### 5.1 The positive effect of proactive personality on students’ knowledge sharing

Proactive personality, as a positive personality trait, exhibits the capacity to positively forecast students’ propensity for knowledge sharing, thereby substantiating hypothesis 1. According to the active motivation model, the interplay between environmental factors and individual traits can influence an individual’s proactive motivation and behavior, with individuals possessing a high level of proactive personality being more prone to benefiting from environmental support (Parker et al., 2010). Previous research has consistently demonstrated that individuals exhibiting a high degree of proactive personality are more inclined to actively engage in and foster developmental endeavors, proactively prepare for the future, and assume greater responsibilities (Major et al., 2006). Furthermore, students possessing a high degree of proactive personality are more inclined to be positively influenced by environmental factors such as teacher autonomous support (Zheng et al., 2020). Consequently, they may experience reduced constraints from their environment and display greater adaptability and proactive utilization of available resources. Studies involving students have revealed the substantial impact of proactive personality on various out-of-role behaviors, including innovative behavior, learning engagement (Bao et al., 2022), and the promotion of knowledge sharing (Zhang and Yang, 2017). Consistent with the demand-resource theory of work, proactive personality as a work resource can help individuals achieve work goals and improve their in-role and out-of-role performance. Consequently, cultivating and exploring students’ proactive traits assumes great significance in fostering knowledge sharing, as well as personal and university knowledge management.

### 5.2 The mediation role of class climate

The findings of this study show that class climate plays an intermediary role between proactive personality and knowledge sharing behavior of college students, which verifies hypothesis 2. The class climate in this study can also be regarded as a specific and in-depth organizational climate. The organizational climate holds a significant sway over individuals’ subjective perception of the objective environment, as well as their attitudes and behaviors displayed within the organizational setting. Bock et al. (2005) assert that an organizational climate characterized by innovation, fairness, and affinity significantly enhances knowledge sharing among members. While an climate of initiative and mutual trust plays a crucial role in promoting knowledge sharing among individuals within an organization (Goh, 2002). Furthermore, individuals with high initiative exhibit strong self-disclosure, positive emotions, and effective interpersonal adaptation (Thompson, 2005), enabling them to establish close and harmonious relationships and foster trust and cooperation. Effective communication facilitates the exchange of work experience and professional knowledge, leading to the sharing of resources. In the context of higher education institutions, the class serves as a one of the most frequent venues for student communication. A favorable class environment offers students a heightened sense of autonomy and fosters a positive and nurturing climate, thereby facilitating deepened communication, mutual learning, collaborative growth, and increased opportunities for knowledge sharing among students.

### 5.3 The mediation role of learning engagement

The findings of this study demonstrate that learning engagement serves as a mediating variable between proactive personality and knowledge sharing behavior among students, which verifies hypothesis 3. It is evident that the proactive personality has a multifaceted influence on students’ knowledge sharing behavior. Specifically, it not only impacts their knowledge sharing behavior through the class climate but also influences knowledge sharing behavior through the intermediary mechanism of learning engagement. The observed positive relationship between proactive personality and learning engagement in this study aligns with the findings of prior research (Zhang and Yang, 2017). Furthermore, it is noteworthy that learning engagement further facilitate the individual’s knowledge sharing behavior. The mediating role of learning engagement between proactive personality and students’ knowledge sharing can be attributed to two underlying factors. Firstly, individuals with proactive personality traits demonstrate active self-motivation to invest in learning resources. Knowledge sharing, as a positive learning strategy, promotes collaboration, effective problem-solving, and prevents the loss of valuable learning resources. The Conservation of Resources theory (COR) (Hobfoll, 1989) emphasizes that individuals can only prevent resource loss by investing the necessary internal resources (Fan et al., 2020). Hence, students must take the initiative to invest resources in order to cope with academic pressure and mitigate the risk of resource loss. Evidently, individual learning investment encompasses a substantial initiative component. Proactive personality, serving as a favorable trait, acts as the catalyst propelling individual initiative. For instance, individuals with high levels of proactive personality proactively influence their environment and actively overcome obstacles to acquire additional resources for problem-solving (Bateman and Crant, 1993). Research in the field of learning further reveals that individuals exhibiting high levels of initiative display more active learning behaviors and exhibit heightened engagement in the learning process (Dong and Liu, 2016). Secondly, individuals possessing elevated levels of proactive personality traits exhibit a proclivity to leverage diverse channels for the purpose of exhibiting their aptitudes. Increased learning investment enhances individuals’ identification with their learning roles, predisposing them to demonstrate their competencies within the academic domain. Academic prowess is primarily demonstrated through the exhibition of scholarly achievements and the dissemination of knowledge. Within this context, knowledge sharing plays a pivotal role as the preferred avenue for proactive individuals to exhibit their competencies. Accordingly, students that display high levels of proactive personality evince a heightened inclination toward and enhanced enthusiasm and conduct with regards to the dissemination of knowledge.

### 5.4 The chain mediating role of class climate and learning engagement

Class climate and learning engagement play a chain mediating role in the influence of proactive personality on knowledge sharing behavior of college students, the hypothesis 4 held true. That is, class climate has a dual influence on students’ knowledge sharing behavior. On the one hand, it serves as a direct predictor of knowledge sharing behavior. On the other hand, it indirectly promotes knowledge sharing behavior by enhancing students’ learning engagement. Drawing from the conformity theory in social psychology, class members’ perception of the class climate, encompassing fairness, support, cohesion, and healthy competition, can elicit a conformity effect, ultimately heightening students’ level of learning engagement. Students characterized by proactive personality traits possess the capacity to enhance their level of learning involvement within a positive class climate. These proactive individuals not only actively participate in learning activities but also contribute to the enhancement of the class climate through proactive knowledge sharing.

## 6 Implications, limitations and conclusions

### 6.1 Practical implications

As the core link of school knowledge management, knowledge sharing is the key to realize the accumulation and innovation of school knowledge resources, reduce students’ learning pressure and improve college students’ professional ability. The act of knowledge sharing fundamentally represents individuals’ active engagement in applying their knowledge. Thus, investigations into knowledge sharing must consider the subjective factors of individuals. The originality of this study stems from its focus on proactive personality as a driver of knowledge sharing, combined with the novel exploration of the mediating effects of class climate and learning engagement. While previous research has investigated how personality traits affect knowledge sharing, studies rarely consider the role of the classroom environment and students’ emotional and behavioral engagement in this process. By integrating these three key variables-proactive personality, class climate, and learning engagement-this research offers a unique perspective on the factors that contribute to students’ willingness and ability to share knowledge. Compared to other studies in the field, this study stands out in its conceptual framework and the emphasis on classroom dynamics. It extends existing theories by suggesting that the influence of personality on knowledge sharing is not direct, but is mediated through the students’ interaction with their learning environment and their engagement in the learning process. This multi-dimensional view adds depth to the existing literature on knowledge sharing in educational contexts. This study holds substantial theoretical and practical implications, thereby warranting its significance.

This work provides empirical evidence for the role of proactive personality in promoting knowledge sharing, while also demonstrating that fostering a proactive personality among students, along with creating a positive class climate and encouraging learning engagement, can be an effective strategy for promoting knowledge sharing in educational settings. In educational practice, ways of implementation, types of activities, involvement of educational support services are the key factors that affect educational effects and learning outcomes. First, universities must recognize that cultivating a proactive personality is an important asset to students’ individual learning. Encouraging students to actively seek knowledge and share learning experiences can enhance their learning experience. For example, universities can hold regular seminars to help students recognize and develop proactive behaviors; Establish mentoring programs that encourage students to take the initiative to help their peers, thereby creating a collaborative and win-win learning environment. Secondly, this study finds that a good class atmosphere is an important factor affecting college students’ knowledge sharing. The more positive the classroom atmosphere, the higher the level of knowledge sharing among students. Therefore, colleges and universities should be committed to creating a positive classroom culture and a good learning atmosphere. At the same time, universities should also provide timely positive feedback and incentives to students who exhibit positive learning behaviors, while providing appropriate guidance to help them build a healthy and positive academic outlook and sense of accomplishment. In terms of the types of activities, universities can regularly organize thematic group discussions and debates to further promote the formation of a cooperative learning atmosphere. Inquiry-based learning (IBL) is a learning method that emphasizes students’ autonomous questioning and independent research, which directly relies on students’ initiative. IBL not only encourages independent learning, but also provides students with rich opportunities for knowledge sharing and collaborative exchange. Educational support services play a vital role in promoting the development of students’ proactive personality traits and creating an environment for knowledge sharing. Through personalized counseling and mentoring programs, students are able to receive targeted guidance that encourages them to develop a proactive mindset. For example, tutors can help students set learning goals, develop learning plans, and provide effective learning management strategies. In addition, investing in the professional development of educators and upgrading the teaching skills of teachers can help them create a more positive classroom atmosphere and promote student engagement in learning. Teachers are specifically trained to identify and foster proactive behavior in students, thereby creating a learning environment that encourages students to actively participate and share knowledge. At the same time, universities can work with architectural design experts to design learning Spaces that meet modern teaching needs, such as interactive learning centers or collaborative learning areas, which can facilitate informal interaction and knowledge exchange among students. Finally, student support services, such as academic counseling and team activities, also help ensure that students are actively involved in the academic journey. Providing extra-curricular activities related to learning not only increases student engagement, but also builds a vibrant and supportive learning community for students. Through the above measures, universities can effectively enhance students’ learning enthusiasm, cooperation awareness and knowledge sharing ability, thus promoting the overall improvement of education quality. Specifically, in the ways of implementation.

### 6.2 Limitations and implications for future research

This study has certain limitations that future research should address. Due to the limitation of research resources, this study issued questionnaires through the Questionnaire Star network, which could not completely select survey objects according to the research design, and the categories of survey objects were not comprehensive enough. In addition, a limiting factor is the lack of information about the distribution of students in different disciplines or majors. This information can be an important issue because students’ perceptions and attitudes can vary by subject or specialty. Another limitation is that college students have not yet achieved complete independence. This study does not involve material/conditional resources in the COR theory, but it should not be ignored that they still live in the family, social, and learning environment. Follow-up studies and comprehensive studies can be used to further investigate these issues in the future.

## 7 Conclusion

This study takes the knowledge sharing behavior of Chinese college students as the research object, confirming that proactive personality significantly influences students’ knowledge-sharing behaviors. However, this relationship is not direct; rather, it is mediated by class climate and learning engagement. Specifically, our findings show that students with a more proactive personality are more likely to engage with the learning process and contribute positively to the class climate, which in turn enhances their willingness to share knowledge with peers. By highlighting the mediating effects of class climate and learning engagement, our study provides a deeper understanding of the processes that link personality traits to knowledge-sharing behaviors. These results contribute to a deeper understanding of the underlying mechanisms governing knowledge sharing dynamics among college students.

## Data Availability

The raw data supporting the conclusions of this article will be made available by the authors, without undue reservation.
